# Recent Progress of Microwave-Assisted Synthesis of Silica Materials

**DOI:** 10.3390/nano10061092

**Published:** 2020-06-01

**Authors:** Borja Díaz de Greñu, Ruth de los Reyes, Ana M. Costero, Pedro Amorós, Jose Vicente Ros-Lis

**Affiliations:** 1 Inorganic Chemistry Department, REDOLí Group, Universitat de València, Burjassot, 46100 Valencia, Spain; borja.diaz@uv.es; 2 Microbiotech SL, 46191 Villamarxant, Spain; ruthdelos@microbiotech.es; 3 Instituto Interuniversitario de Investigación de Reconocimiento Molecular y Desarrollo Tecnológico (IDM), Universitat Politècnica de València, Universitat de València, Doctor Moliner 50, Burjassot, 46100 Valencia, Spain; ana.costero@uv.es; 4 Institut de Ciència dels Materials (ICMUV), Universitat de València, P.O. Box 22085, 46071 Valencia, Spain; pedro.amoros@uv.es

**Keywords:** microwave assisted synthesis, silica, mesoporous, nanoparticles

## Abstract

Microwaves are a source of energy of great interest for chemical synthesis. Among nanomaterials, few are as versatile as silica—it forms mesoporous materials and nanoparticles, it can be incorporated as shells or loaded in composites, it can also be functionalized. Despite the relevant properties of silica, and the advantages of the use of microwave as energy source, its use in silica-based materials is not frequent. We report herein a compilation of the research results published in the last 10 years of microwave assisted synthesis of silica based materials. This review includes examples of mesoporous materials for waste removal, catalysis, drug release, and gas adsorption applications, together with examples based in the optimization of the synthesis conditions. In the case of non-porous materials, examples of analytical applications, coating of metallic nanoparticles, and SiO*_x_*-C materials have been collected.

## 1. Introduction

Silicon oxides have outstanding features, such as good stability, ease of functionalization, biocompability, etc. [1]. In this way, silica solids have been widely applied to multiple applications. Since the discovery of MCM-41 by Mobile Oil Corporation in 1992 [2], many mesoporous silica materials have been reported [3]. Between these materials, the most common families are those referred as M41s (e.g., MCM-41 and MCM-48), and SBA-type (e.g., SBA-15 and SBA-16). These mesoporous materials can be produced simply by varying the surfactant used as template during the synthesis, and its concentration [4]. Other synthesis parameters—such as temperature, time, type of reagents, and their molar ratio—also affect to some extent certain features of the final material produced, like size and shape of the particles. Their structural properties provide the materials of large surface areas and pore diameters from 2 nm up to 50 nm and can be classified as nanomaterials according to IUPAC definition [5]. Silica mesoporous nanomaterials have been used as supports for catalysis [6], biological applications [7], sensors [8], or to adsorb and remove heavy metal ions from solution [9]. However, not only silica is important in these applications, but also the presence of heteroelements or anchored organic groups which give different functionalities to the materials.

Synthesis conditions for producing silica materials with controlled parameters—such as size, shape, or porous structure—are presently well-established [10,11]. However, most of the published procedures are time intensive, because they are produced under conventional heating and the nucleation-growing and ageing processes requirements can range from hours to several days. In order to solve this problem, microwaves emerged as a feasible alternative to notably reduce synthesis times, yielding reproducible solids with even improved properties compared to those obtained under conventional conditions [12,13,14,15].

Microwaves are electromagnetic waves with frequencies in the range of 300 MHz–300 GHz, which corresponds to wavelengths between 1 m and 1 mm respectively. Despite the low energy associated to these values, they are used worldwide to transfer information and energy (heat generation). Microwave heating has already been thoroughly discussed by other authors [16,17,18]. When polar and charged molecules are subjected to this kind of electromagnetic radiation, they try to align with the electromagnetic field. As this is an oscillating field, molecules become extremely agitated, producing the dissipation the energy of the radiation and the increase of the inner temperature of the object subjected to microwaves. Many susceptor materials have been developed to efficiently convert microwaves into heat, and different composites with improved features, such as resistance to oxidation, has been produced [19,20]. As a result, the interaction of microwaves with matter produces a temperature gradient profile that is inverted compared to conventional heating [21]. Conventional heating methodologies—such as oil baths, heat-on blocks, or heating mantles—transfer the heat through convective thermal gradients from the surface to the inner material or solution. Therefore, the surface of the container reaches a much higher temperature than the other parts of the system, causing a non-homogeneous heating that can led to product or reagent decomposition as well as the formation of undesired by-products, decreasing reaction yield and making more necessary the subsequent purification steps [22]. Alternatively, microwaves are able to penetrate non-metallic materials to a depth that depends on the dielectric properties of the material and to heat them up homogeneously from inside. Consequently, many advantages related to microwaves have been reported in research: reduced reaction times, higher reaction yields, and selective heating among others [23]. All these advantages have led to the development of new technologies in fields as diverse as food processing [24], water treatment [25], drying [26], or chemical synthesis [27]. These applications have been supported by parallel research into specific technologies of microwave devices, adapted to the specific needs of each one of them [28].

Focusing on chemical synthesis, microwaves have been applied not only to organic reactions [22] but also to inorganic products [29]. In this way, many materials like metal oxides [27], metallic nanomaterials [30], metal organic frameworks (MOFs) [31], polymers [32], or graphene-based materials [33] synthesized by microwave-assisted techniques have already been reviewed. Although some reviews including the synthesis of microporous and mesoporous materials (including silica) by microwave irradiation have been published [34,35,36,37], no specialized reviews targeting the synthesis of silica materials with this source of energy have been reported up to date. This review focuses on the full description of silica materials produced under microwave-assisted techniques during the last decade, more specifically on mesoporous and non-porous materials, and their applications.

## 2. Microwave-Assisted Synthesis of Silica Mesoporous Materials

Thanks to the good properties of mesoporous silica materials commented above, they have been used in multiple applications and prepared in different ways. Among these possibilities, the hydrothermal route is the most commonly reported in bibliography. Hydrothermal synthesis can be defined as the aqueous chemical synthesis reactions taking place in a sealed reactor above ambient temperature and pressure [38]. However, this term expanded its use, and nowadays refers also to reactions taking place in organic solvents, that should be named as solvothermal [39], or even in open reactors at relatively low-temperature/pressure conditions [40]. With this open-concept in mind, a variety of this one is the microwave-assisted hydrothermal route, which heats the solution under microwave irradiation in order to save time compared with conventional methodology. A description of the synthesis procedures of silica mesoporous solids prepared with microwaves together with the most remarkable applications are reported below.

### 2.1. Waste Removal Applications

Porous materials have been widely applied to the extraction of contaminants, either organic or inorganic, from different sources. Sorbents must fulfill several characteristics: good stability, large surface area and pore volume, and high sorption selectivity towards desired species, among others. Silica porous solids join all these benefits together. Besides, pore channels are adjustable and can be easily functionalized, giving improved selectivity and extraction capability.

According to the silica source employed during the synthesis, solids can be divided into conventional or non-conventional types. In recent years, non-conventional silica sources have emerged as a feasible alternative for the recovery and valorization of industry by-products. These methods are able not only to produce porous materials but also to apply them for the removal of other pollutants.

#### 2.1.1. Mesoporous Materials from Conventional Silicon Sources

Silicon sources such as tetraethoxysilane (TEOS) or sodium silicate are among the most common ones used for nanomaterials production based on silica. Many solids have been prepared by using these two reagents due to their cost, stability and suitable hydrolysis rate. All these features together, apart from well-defined synthesis conditions, allow the reproducible preparation of high-quality materials that have been employed in countless applications, like pollutant removal or pre-concentration of species of interest.

A first approximation to apply mesoporous materials to the removal of contaminants can be addressed to carbon dioxide. CO_2_ is a greenhouse gas whose emission makes up more than 75% of the greenhouse gases, making it the main responsible of global warming and climate change. In addition to reduce combustion of fossil fuels, CO_2_ capture and sequestration (CCS) technologies have been investigated as an alternative to achieve global carbon reduction emissions, a goal where mesoporous silica materials have been also implemented [41]. Mesoporous silicas for CO_2_ capture were prepared by optimizing pore length and further modifying the solids with different amines. Amine modifications allowed to adsorb CO_2_, while shortened pores gave faster adsorptions and relatively higher CO_2_ retention capacity. In this way, the authors prepared a hexagonal short-pore length SBA-15 (SSBA-15) via an energy-efficient process under microwave-hydrothermal conditions by using TEOS as silicon source and copolymer P123 as the structure directing agent. Pore length was controlled by varying durations of ripening step (stirring at low temperature) and aging step (microwave-hydrothermal treatment). Thanks to the use of microwave energy, synthesis time was reduced from 48 h for conventional hydrothermal treatment to 2 h. The influence of aging and ripening time to the size and shape of pores and particles were studied through electronic microscopy, XRD and N_2_ adsorption/desorption isotherm techniques, showing how aging duration had a greater impact on the final solids obtained. 2 h ripening and 2 h aging was found out as optimal conditions. Under these parameters, cuboid-like particles of SSBA-15 with pore length ca. 450 ± 50 nm and pore diameter around 10 nm were obtained. These solids showed a highly ordered 2D hexagonal symmetry, with a narrow pore size distribution. Finally, modification of SBA-15 with amines led to an optimal CO_2_ capture of 1.86 mmol g^−1^ when triethylenetetramine (TETA) was selected.

With these results in mind, Liu et al. obtained polyamine-modified mesostructured silica adsorbents by using a simple one-pot method under microwave irradiation [42]. Several polyamine types and load amounts were tried in order to optimize CO_2_ capture. A SBA-15 solid, obtained under conventional conditions by the post-synthesis impregnation method, was also prepared for comparative purposes. According to X-ray diffraction (XRD) profiles, samples prepared under microwave-assisted heating showed mesostructured characteristics, but lacking a long-range order. This is most probably due to the successful incorporation of polyamines to the structures. This modification is further justified by the presences of infrared absorption bands attributed to characteristic vibration bands of the amine groups (–NH_2_) and amine species (–RNH_2_). Regarding textural properties, N_2_ adsorption/desorption isotherm measurements reported type III isotherms with H3 hysteresis loops. Brunauer–Emmett–Teller surface area (*S_BET_*) and total pore volume (*V_Tot_*) increased compared to conventional SBA-15 synthesized by hydrothermal method. Finally, CO_2_ uptake capacity was measured, obtaining a maximum value of 4.43 mmol g^−1^ when the solid was synthesized under microwave conditions with tetraethylenepentamine.

Apart from inorganic pollutants, like CO_2_, mesoporous solids have been also employed for the removal of organic species. Among all the organic pollutants, dye wastewaters produced by textile, ink and paper manufacturing industries are an important environmental pollutant. In particular, methylene blue (MB) is probably the most common one. MB is usually selected as target in order to check the ability of new methodologies able to remove organic species. In this way, mesoporous materials have been employed not only to directly adsorb MB on their surface, but also as a support material of photocatalyst like TiO_2,_ able to degrade organic pollutants under irradiation of UV light. In this way, SiO_2_-TiO_2_ nanoparticles were synthesized by the sol–gel method using TEOS and TEOT (Ti(OC_2_H_5_)_4_) as silicon and titanium sources respectively, and stearyltrimethylammonium chloride as a template agent [43]. By the addition of acetylacetone as the capping agent for Ti alkoxide, the hydrolysis rate was reduced and Ti was homogeneously distributed within the mesoporous SiO_2_ matrix at Ti:Si ≤ 0.1. Solids were synthesized in just 30 min under microwave heating at 60 °C, showing spherical shape and particle sizes distributed between 0.1 and 20 µm, with a pore size of 3.4 nm. An increase in Ti:Si molar ratio gave irregular morphologies of particles. The spherical solids obtained showed a moderate activity towards MB degradation.

Xu et al. improved the degradation efficiency of MB with SiO_2_-TiO_2_ nanoparticles [44]. In this case, the authors prepared mesoporous solids under a two-step procedure. First, silica microspheres were produced by microwave hydrothermal synthesis for 90 min at 120 W, giving uniform smooth-surfaced microspheres of 1.3 µm size. Then, the solids were doped with TiO_2_ particles in a post-grafting step, producing rough surfaces but keeping the uniform pore structure of initial silica mesoporous samples. As a consequence of TiO_2_ modification, BET surface area halved from 653 to 299 m^2^ g^−1^. Ti was not homogenously distributed in the silica matrix, but present on the surface of the spheres as TiO_2_ in both anatase and rutile phases. Thanks to the presence of these phases, discoloration of methylene blue was greatly enhanced, especially when alkaline pH and UV light were applied.

Apart from MB, the degradation of other organic compounds has been tried. In this way, mesoporous Ti-MCM-41 nanoparticles were synthesized by a microwave-assisted method, and their photocatalytic activity was tested for the oxidation of dibenzothiophene with H_2_O_2_ [45]. For the synthesis, H_2_O_2_ was added to tetrabutylorthotitanate (TBOT), and cetyltrimethylammonium bromide (CTAB) and TEOS worked as template and silicon source respectively. After 1 h stirring, the mixture was hydrothermally heated in a household microwave in increasing time intervals (total irradiation time: 41 min) at a low power (210 W). Thanks to the fast and homogeneous heating provided by microwaves, small particles in the range of 4–8 μm were produced. Ti was homogenously distributed within the silica structure, causing a slight decrease in porosity compared to those parameters obtained under conventional heating. In any case, BET surface area and pore diameter were very similar to those typical of unmodified MCM-41. Finally, its photocalytic activity was checked against the oxidation of dibenzothiophene with H_2_O_2_, observing no remarkable differences between solids produced under conventional or microwave heating.

Other metal oxides like CeO_2_ have showed outstanding properties towards organic pollutant degradation. CeO_2_-SiO_2_ nanoparticles were synthesized by microwave-assisted irradiation method using a mixture of CTAB, cerium nitrate, and TEOS under acidic media at 160 °C for different times (30–180 min) [46]. Although longer reaction times gave more crystalline solids, the highest catalytic activity for MB degradation was observed for the sample produced with the shortest reaction time (30 min), because longer times caused the surface area to decrease. For 30 min irradiation time, CeO_2_-SiO_2_ nanocube shape solids with size of about 8 nm were prepared. This solid showed a type IV N_2_-adsorption isotherm, with a high BET surface area of 335 m^2^ g^−1^. Compared to conventional method, hydrothermal treatment required longer reaction times (24 h) to give aggregated spherical nanoparticles of 15 nm size with lower surface area (90 m^2^ g^−1^). Finally, photocatalytic activity of the CeO_2_-SiO_2_ nanoparticles prepared by 30 min irradiation with microwaves was faced with TiO_2_ P25 (anatase phase, used as commercial reference). It was found to better degrade MB dye under UV light than the standard TiO_2_ photocatalyst, most probably because of the higher surface area of CeO_2_-SiO_2_ (335 m^2^ g^−1^) compared to that of P25 (50 m^2^ g^−1^).

Periodic mesoporous organosilicas (PMOs) have been also used for photodegradation of methylene blue. PMOs are composed by an organic-inorganic framework in which organic groups are uniformly distributed within the material walls, while leaving the porosity free. A PMOs containing tetrakis(carboxyphenyl)porphyrin (TCPP), denoted as TCPP-PMO(II), was synthesized by self-assembly of TCPP-tetrasilane together with sodium metasilicate under acidic conditions in presence of the surfactant Pluronic P123 [47]. Microwave irradiation at 100 °C for 2 h yielded the hybrid organic–inorganic material as a platelet aggregation, with a surface area of 493 m^2^ g^−1^ and a pore diameter around 8.7 nm with hexagonal shape. As expected for a PMO, TCPP units were included in the silica wall, and thus an increment in TCPP loading produced worse mesostructural features. Finally, the synthesized TCPP-PMO(II) was applied not only to MB degradation but also to hydrogen transfer reactions, and further modified with Fe to catalyze the oxidation reaction of cyclohexene.

As a special type of materials, the use of silica containing magnetic nanoparticles for extraction purposes in analytical studies has emerged as a very dynamic research area. These materials are comprised of a mesoporous shell on the surface of a magnetic carrier. The silica mesoporous shell can concentrate pollutants on their surface, and the material can be easily recovered from media by simply applying an external magnetic field to the solution. This allows conducting environmental studies of organic and inorganic substances present in trace amounts. Microwave synthesis becomes a simple and fast method for synthesizing such materials.

**Scheme 1:**
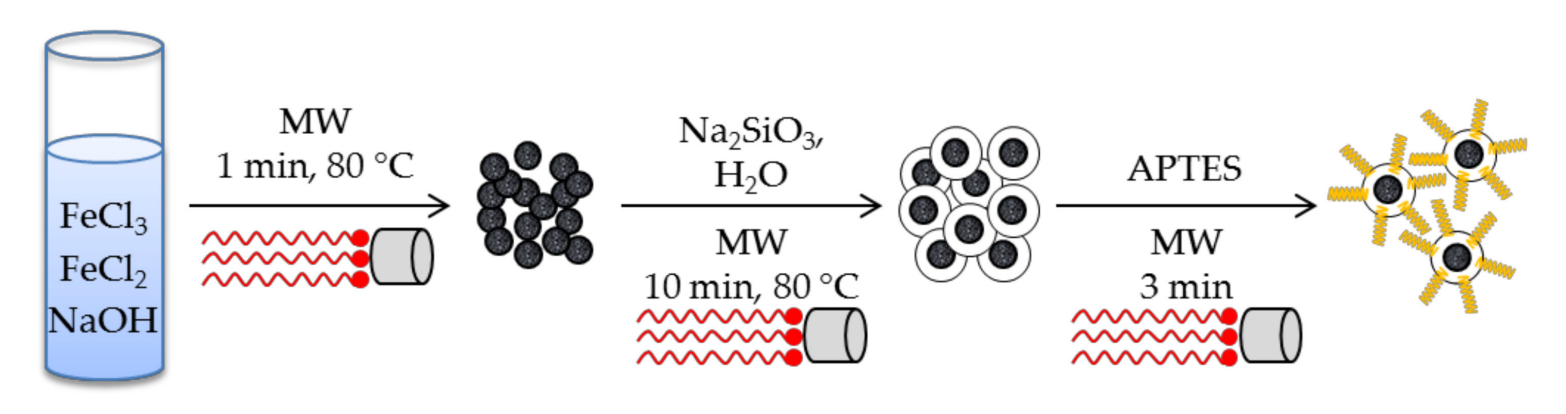
Synthesis of [Nano-Fe_3_O_4_@SiO_2_@NH_2_] solid under microwave irradiation [48]. Note how MW have been used in every step of the synthesis.

Nanomagnetic iron oxide functionalized with 3-aminopropyltriethoxysilane (APTES) have been synthesized using a microwave-assisted procedure [48]. After three successive microwave-assisted heating procedures, in which the core, the shell and the surface modification were respectively accomplished, the [Nano-Fe_3_O_4_@SiO_2_@NH_2_] solids were obtained (Scheme 1). Silica shell was obtained within 10 min by simply placing magnetite nanoparticles in water with sodium silicate in a microwave at 80 °C, giving homogeneous particles size distribution in the range of 19–44 nm according to SEM. Although no surfactant was added during the synthesis, the solid showed a BET surface area of 308 m^2^ g^−1^, high enough for extraction applications. After functionalizing the solid with APTES, under microwave irradiation for 3 min, it was applied to the extraction of heavy metal ions (Pb(II), Cu(II), Cd(II), and Hg(II)).

In another approach, a [Fe_3_O_4_@TEOS@CTAB@TEOS + MPTEOS] solid was obtained by sequential microwave-assisted reactions [49]. Based on several layers, the latest ones were porous thanks to the presence of CTAB. Moreover, the mixture of tetraethoxysilane (TEOS) and (3-mercaptopropyl)triethoxysilane (MPTEOS) on the surface allowed the simultaneous or sequential pre-concentration of components of various natures. In particular, the material was successively applied to the pre-concentration and subsequent determination of two groups of pollutants: phenols (of various nature) and heavy metals (Рb(II) and Сd(II)).

#### 2.1.2. Mesoporous Materials from Non-Conventional Silicon Sources

**Scheme 2:**
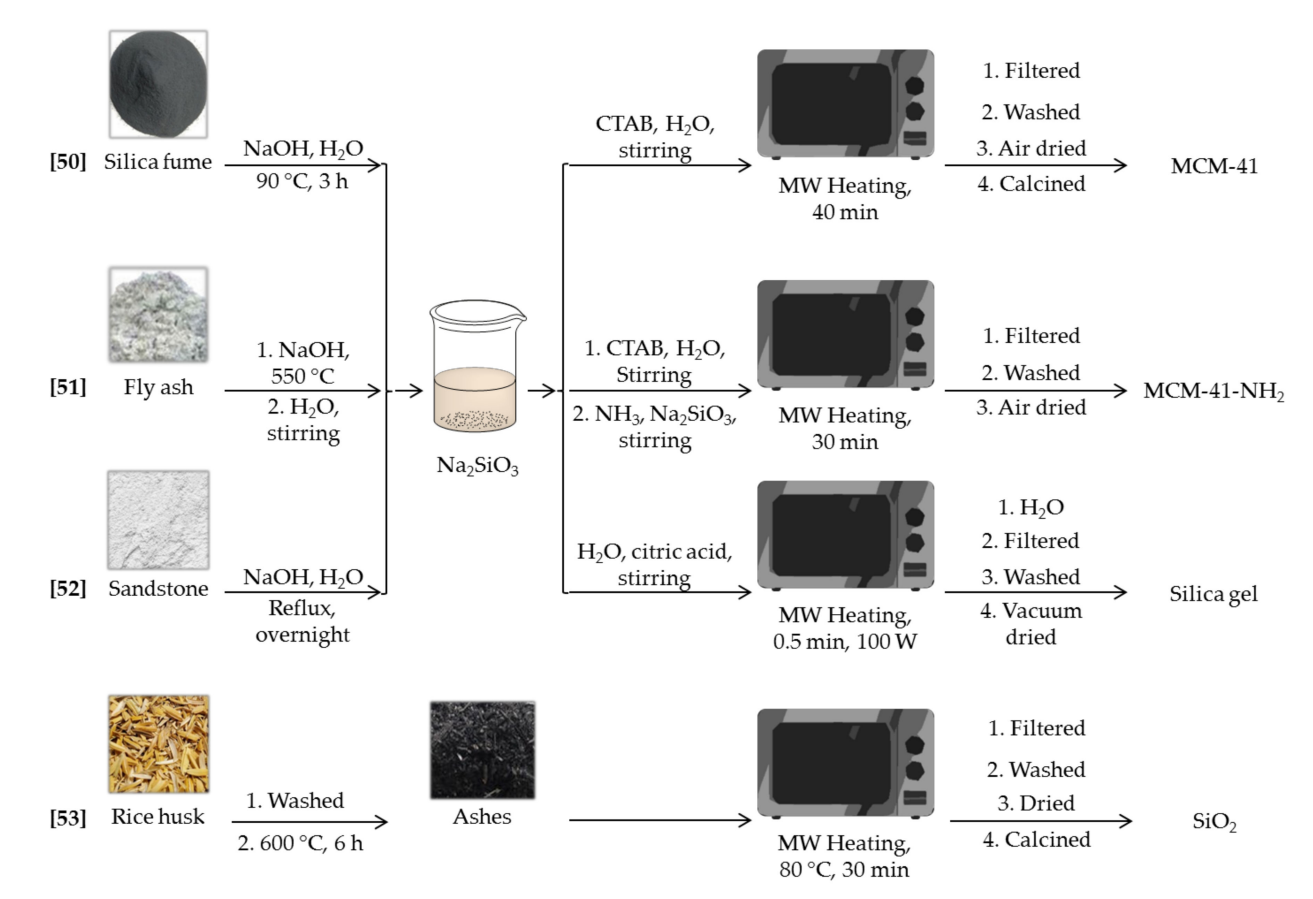
Synthesis scheme for the microwave assisted synthesis of silica materials [50,51,52,53] by using non-conventional silicon sources.

Despite the widespread use of tetraethoxysilane (TEOS) and sodium silicate as siliceous sources for the preparation of silica mesoporous materials, there is a growing interest in the search for, and application of, alternative silica precursors. Cheap silicon sources, like fly ashes or agricultural wastes, have attracted an increasing attention in order to reduce the high cost of the materials. This is a key aspect for applications requiring large scale materials. Moreover, they can be considered as a green way to reuse industrial byproducts. A summary of the materials and the experimental conditions can be found in the Scheme 2.

For instance, MCM-41 type mesoporous materials were synthesized by microwave heating using silica fume, a very fine amorphous silica powder obtained as a byproduct during metal production processes [50]. With a SiO_2_ content around 85 wt%, silica fume was dissolved in basic media to give a sodium silicate solution, which was directly used during the MCM-41 synthesis. The effects of microwave heating times and acids, used to control the pH, on the pore structure were studied. In short, reaction times of 40 min and addition of citric acid gave big particles with MCM-41 structure. Longer reaction times produced a less ordered hexagonal mesostructure, whereas the use of inorganic acids caused pore volume and pore diameter to decrease. Once characterized, samples were evaluated as adsorbents for the removal of Cu(II), Pb(II), and Cd(II) cations from aqueous solutions, showing good performance and a maximum adsorption capacity of 36.3, 58.5, and 32.3 mg g^−1^ respectively.

In another approach, MCM-41-NH_2_ was synthesized from fly ash [51]. Fly ash is a solid waste of power industry, generally composed of silicon and aluminum oxides, with SiO_2_ content above 50 wt%. When calcined in a muffle with NaOH, the resulting powder can be used as silicon source. A filtered solution of this solid, along with CTAB and ammonia solution, was microwave irradiated for 30 min to yield MCM-41-NH_2_. Thanks to the presence of ammonia solution, the whole solid surface and pores were functionalized with amine groups. Consequently, the degree of crystalline order was lower than that for typical MCM-41 solids, but surface area (258 m^2^ g^−1^) was good enough for allowing Cr(VI) removal. Authors could verify the important role that amino groups play in the adsorption process. They do not only absorb Cr(VI) on the surface, but further reduce it to Cr(III), minimizing its toxicity.

Sandstone can be also used as raw material for the synthesis of silica nanoparticles [52]. In this case, silicon content is much higher than in previous materials (it ranges from 98.5 to 99.6 wt%). After solving the sand in basic media, the resulting sodium silicate solution was adjusted to pH 7 with citric acid solution and then irradiated with low power microwaves to produce silica solids. Times as low as 30 s produced interconnected particles, whose sizes are within the 16–28 nm range. The spaces between particles were responsible of the textural features, which were improved reducing the aging time. Although surface area was relatively small in all cases, around 200 m^2^ g^−1^, the solids produced were applied to the adsorption of alkaline earth metals from aqueous solutions. The gels showed high efficiency for Sr(II) and Ba(II) adsorption, but this capability dramatically decreased in presence of Mg(II) and Ca(II), due the higher affinity for the latest two cations.

Adsorption of organic species, like methylene blue, has been also studied using other non-conventional siliceous sources [53]. Rice husk, an agricultural waste, was employed as precursor for the synthesis of silica nanoparticles. After burning the husks, the ashes were leached in a microwave oven for 30 min along with HCl to yield amorphous silica nanoparticles with a mean particle size of 93 ± 5 nm. As no surfactant was employed during the synthesis, porosity of the samples was very limited, with a BET surface area of just 84 m^2^ g^−1^. These particles were applied to the study of the adsorption of MB from aqueous media, fully studying kinetics and thermodynamics processes taking place through different adsorption models.

A summary of the literature discussed in this section, regarding the synthesis of silica nanoparticles for waste removal applications, is shown in Table 1.

### 2.2. Catalysis

Thanks to their large surface area and easy surface functionalization, mesoporous silica nanoparticles have been used as support of different catalysts used in a broad range of chemical reactions, like the reported examples with TiO_2_ for the photodegradation of different pollutants or the solids modified with Fe to produce oxidation reactions [43,44,45,47]. Park et al. developed plugged templated silica for asymmetric catalysis [54]. This material had a triple functionality which enabled organic asymmetric reactions to take place. The material was structurally comprised of open and plugged pores functionalized with the chiral amino-acid L-proline, which acted as organocatalyst. The material offers excellent properties to be applied as catalysts in the asymmetric addition reaction of diethyl malonate and in the epoxidation of α,β-unsaturated aldehydes. Regarding the synthesis conditions, a mixture of L-proline, sodium metasilicate, and Pluronic P123 as template was placed under acidic condition and aged by direct microwave irradiation at 100 °C for 2 h. As a result, the plugged pore structure having L-proline functionality was obtained. The material shows a hexagonal mesostructure with uniform cylindrical channels with a pore length around 300–400 nm. By comparison, a post synthesis L-proline functionalized SBA-15 gave no pore plugging effect. Although L-proline load was higher than in the directly charged solid, it showed worse catalysis effect.

SBA-15 solids with Ti included in the silica framework (Ti-SBA-15) were synthesized by microwave-assisted method in order to check their behavior in hydrodesulfurization and hydrodearomatization reactions [55]. The material was further modified including CoMo catalysts on its surface. The mixture of TEOS, titanium isopropoxide, P123, and ethanol was aged by microwave-hydrothermal method in the presence of water for 1 h at 90 °C. A final sieving of the solids by extrusion molding method afforded 425–800 μm particles. Ti was successfully incorporated into the SBA-15 structure frame, without affecting the basic framework of SBA-15. No presence of rutile or anatase phases was detected by XRD or diffuse reflectance UV-vis. However, Ti had a great influence in the behavior of the final solid loaded with CoMo catalyst, increasing hydrodesulfurization reaction activity but decreasing hydrodearomatization.

Titanium has also been introduced into MCM-41 silica framework for photocatalysis applications [56]. Different Ti contents, from 1 to 12 wt%, were prepared, and then they were microwave irradiated at 120 °C for 40 min to obtain Ti-MCM-41 samples. As a result, titanium species entirely incorporated into the solid framework, no matter what Si:Ti ratio was used. However, as Si:Ti ratio decreased, the long-range order of structure diminished. The shape of the isotherms was similar, showing a slight shift toward lower relative pressure when Si:Ti ratio decreased. Thus, Ti induced lower pore volumes and BET surface areas, but in all cases the materials were able to work as efficient catalysts of the transesterification reactions of dimethyl oxalate and phenol.

### 2.3. Drug Release

Mesoporous materials can be also used to keep substances of interest in their pores, therefore serving as a carrier and protecting the load from aggressive environments. This behavior is especially interesting for sensing and/or drug delivery applications. Thanks to the homogeneous heating created by microwaves, solids produced by this methodology have been applied to drug delivery, where small particle sizes and narrow distributions are required. In this way, mesoporous silica nanoparticles for ibuprofen delivery were synthesized by microwave synthesis [57]. Although variable microwave powers were applied (100–450 W), the higher the power the smaller and more crystalline the particles were, producing smaller spherical particles sizes (30–45 nm) with smooth surface. Therefore, irradiation at 450 W for 1 h afforded solids with high crystallinity and high surface areas (817 m^2^ g^−1^). In the case of using a power of 100 W, irregular particles with a rough surface were formed. On its behalf, powers of 500 W produced disordered structures. Finally, behavior of the solids towards ibuprofen adsorption and release was characterized.

### 2.4. Gas Adsorption

Apart from the adsorption of pollutant gases, like CO_2_ already commented [41,42], mesoporous silica nanoparticles have been applied to the adsorption of other gases. A MCM-41 type material was prepared in 30 min under microwave heating, and further impregnated with Pd in order to improve its hydrogen storage properties [58]. Synthesis was tried at two different powers (90 and 120 W) and at two different Pd concentrations (1:100 and 10:100 Pd:MCM-41 weight ratio). Synthesis at 90 W gave smaller pore sizes and lower BET surface areas, but in both cases higher than 1400 m^2^ g^−1^, thus 120 W was selected as optimal power. On the other hand, although impregnation with Pd solution was performed before template removal by calcination, a hydrogen uptake as high as 1.74 wt% at 298 K and 10 bar pressure for 10:100 Pd:MCM-41 synthesized at 120 W was obtained. This value doubled the adsorption capacity of MCM-41 without Pd.

A summary of the literature discussed in Section 2.2–2.4 is shown in Table 2.

### 2.5. No Reported Applications

Mesoporous solids are not always synthesized taking in mind a direct application. In some cases, a thorough study of how different parameters affect the final solid or what steps are involved in the synthetic pathway is, by itself, interesting enough. In this section, several mesoporous solids, in which no application is reported, will be reviewed.

#### 2.5.1. Synthesis Optimization of Siliceous Porous Particles

MCM-41 is one of the most studied and employed mesoporous solids. It has been used in a large variety of applications, including adsorption, sensing, and catalysis among others. Although the material has been previously synthesized by microwave assisted methods [34,59], most of the syntheses were conducted with fixed parameters regarding the siliceous source or the concentration of surfactant or NaOH employed. Wittayakun et al. conducted the microwave assisted synthesis of MCM-41 at different CTAB:SiO_2_ and NaOH:SiO_2_ ratios, and several reaction times [60]. The effect of each parameter was studied successively; comparing the effect produced in X-ray diffraction peaks and pores uniformity. In this way, a CTAB:SiO_2_ mole ratio of 0.3, an equimolar NaOH:SiO_2_ amount and a microwave reaction time of 90 min were selected as optimal conditions. Shorter irradiation times are not recommended, in order to increase the rate of condensation and produce more ordered structures. The solids showed similar properties to those prepared under conventional method, with a BET surface area of 1138 m^2^ g^−1^ and a pore diameter of 3.21 nm, but with an energy saving derived from the use of microwave methods.

The effect of the applied microwave power to the synthesis of MCM-41 has also been studied [61]. Two powers were selected to carry out the reactions: 80 W and 120 W. Reaction temperatures of 90 °C and 120 °C were reached with 80 and 120 W respectively. Thus, their impact on the final solid cannot be blame upon power or temperature independently, but to the combination of both. Furthermore, several microwave irradiation times from 10 to 60 min were tried. In short, higher powers produced thinner walls, but very uniform and ordered pores. Despite wall thickness, all the solids prepared showed good thermal stability according to thermogravimetry. Moreover, reactions at 120 W gave faster reaction rates and more nucleation, producing smaller particles of about 70 nm. With respect to porous properties, irradiation at 120 W for 30 min produced highly ordered structures with a surface area of 1438 m^2^ g^−1^, and a pore diameter of 4.0 nm.

In another article, the interest was not focused on the hydrothermal microwave-assisted synthesis of MCM-41 but in refining a procedure to remove CTAB surfactant by ion-exchange extraction from the mesostructured solid [62]. Regarding the synthesis, the developed method yielded mesoporous MCM-41 solid in 90 min when heated in a microwave at 100 °C. X-ray diffraction peaks, type IV shape of nitrogen adsorption/desorption isotherms, and a BET surface area of 1138 m^2^ g^−1^ evidenced the successful synthesis of this mesoporous solid. Finally, the surfactant extraction was optimized, obtaining the use of NH_4_Cl in methanolic solution at 60 °C for 15 min as the most suitable conditions.

Regarding the surfactant, MCM-41 is normally synthesized using cetyltrimethylammonium bromide (CTAB) as a template. Changing the surfactant leaves a wide variety of mesoporous structures. Furthermore, the properties of the solids can be deeply modified by simply changing the counterion. A procedure for the microwave-assisted synthesis of mesoporous solids by using cetyltrimethylammonium tosylate (CTAT) instead of bromide was developed [63]. These solids were compared with those obtained by conventional heating and/or by employing CTAB as a template. Tosylate was found to be a better counterion than bromide, in the way that hydrophobic template counterions favor the silica growth and allow the synthesis of the final material at higher temperatures and thus in shorter times (190 °C and 2 min, respectively). In any case, solids prepared using CTAT were relatively insensitive to the temperature plateau, with no pore expansion, which reveal that both mass and heat transfer processes were avoided by using short reaction times. Structurally high-quality materials at the gram scale were prepared.

**Scheme 3:**
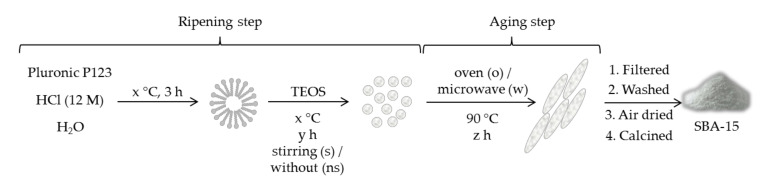
General synthesis scheme for the preparation of SBA-15 [64]. Different ripening temperatures (*x* = 28, 40 or 55 °C), ripening times (*y* = from 2 to 24 h), stirred/unstirred samples (s/ns), aging times (*z* = from 2 to 24 h), and oven/microwave (o/w) conditions were tried. Optimal conditions were found to be *x* = 40 °C, *y* = 2 h, stirring (s), and microwave for *z* = 2 h.

Among other surfactants applied to the microwave synthesis of mesoporous solids, those materials were obtained by using poly(ethylene oxide) (PEO) and poly(propylene oxide) (PPO) triblock copolymers as a template are of special interest. One of these polymers is P123 (EO_40_PO_70_EO_40_), commonly used during the synthesis of SBA-15 mesoporous solids. Although surfactant concentration kept constant, the influence of other synthesis parameters on the characteristics of SBA-15 type solid was studied [64]. Both steps involving the formation of solid particles were studied (ripening and aging), although microwave irradiation was just applied during aging step (Scheme 3). For the ripening step, time, stirring, and temperature were optimized. Regarding aging, heating at 90 °C either in an oven or in a microwave was considered. It was found that silanol content is slightly modified with the ripening temperature, but strongly increases with aging (under microwave) duration. An optimal aging time of 2 h was found. This increased silanol content—together with a higher specific surface area, pore diameter, and total pore volume—improved its sorption properties whatever the duration of the ripening was. However, microwave heating also affected particles morphology, producing wide and large aggregates, but structurally ordered when the ripening duration is 2 h at least.

Another PEO and PPO triblock copolymer is Pluronic F127. In this case, F127 (EO_106_PO_70_EO_106_) is used as a template during the synthesis of SBA-16 type mesoporous solids. The synthesis of this kind of materials has been also accomplished by using a microwave-assisted method [65]. This time, microwaves were applied in the self-assembly and hydrothermal treatment steps. Self-assembly was studied at 40 °C with diverse irradiation times. The influence of time during self-assembly had low impact on the final material, but authors recommend 6 h. In the second step, both temperatures and times were modified. It was found that lower hydrothermal temperatures (100 or 120 °C) produced cage-like mesostructures, while channel-like materials were formed at higher temperatures (140 and 160 °C). Furthermore, the duration of microwave-assisted hydrothermal treatment affected the porosity. Longer times increased the complementary porosity, with an optimal time around 6–9 h. Under these optimal conditions, and with a hydrothermal temperature of 100 °C, materials with a BET surface area of 866 m^2^ g^−1^ and a pore diameter of 9.0 nm were prepared.

The microwave assisted synthesis of Fudan University 1 (FDU-1) silica has also been reported. As well as SBA-15 and SBA-16 synthesis, preparation of FDU-1 requires a PEO-PBO-PEO triblock copolymer as template (EO_39_BO_47_EO_39_). The more hydrophobic character of the polymer promotes in FDU-1 a highly ordered cage-like cubic structure with 12 nm pores, much smaller than reported for conventional SBA-16 solids. Bruns et al. conducted two factorial designs for studying the microwave-assisted synthesis of FDU-1 [66]. In these preparative designs, authors used Vorasurf 504^TM^ (Dow Polyurethanes, Midland, MI, USA), a copolymer of an average EO_38_BO_46_EO_38_ composition, and parameters like acid concentration, stirring time, microwave irradiation time, and microwave temperature were considered. As a result of the study, optimized reaction conditions were reported: 2 M HCl, dissolution in ethanol, 24 h stirring, and 60 min microwave-assisted reaction time at 100 °C. Following these parameters, a FDU-1 solid with a BET surface area of 622 m^2^ g^−1^ and pore size of 8.5 nm could be prepared.

#### 2.5.2. Silica Materials Containing Heteroatoms

The synthesis of silica nanomaterials containing heteroatoms, such as metals, requires longer reaction times in order to achieve a certain compositional homogeneity. Microwave irradiation techniques have been successfully applied to the synthesis of these modified frameworks, achieving a remarkable reduction in the time needed. Indeed, zirconium was successfully introduced into the MCM-41 mesoporous framework by using a microwave-assisted procedure [67]. Several ZrO_2_:SiO_2_ molar ratios solids were prepared. Even at high concentrations no ZrO_2_ segregated phases were detected. Moreover, it was found that mesoporous structure was not altered at low Zr concentrations (up to a ZrO_2_:SiO_2_ molar ratio of 0.05:0.2) but gave disordered phases with the highest Zr contents. BET specific surface area and pore volume decreased as the amount of zirconium increased, whereas the pore sizes gradually increased.

Other elements rather than transition metal ions have been also incorporated. Phosphorus element has been added to the framework of mesoporous solids as phosphonic acid. Ordered SBA-15 type mesoporous silicas with phosphonic acid groups were effectively synthesized under microwave irradiation [68]. Changing the siliceous source produced different particle morphologies: large agglomerates composed of smaller submicrometric particles or particles larger than 1 micron, which are glued together, by using TEOS or sodium metasilicate, respectively. In both cases, the functional group was present. The mesostructure was almost as well defined in the microwave prepared material as that obtained in a conventional oven, with a specific surface area of around 700 m^2^ g^−1^. Therefore, in this case, the major advantages of the MW treatment were the shorter synthesis time and a selectable particle shape.

#### 2.5.3. Hybrid Organic–Inorganic Materials

Benzene bridged PMOs were prepared under microwave-assisted conditions [69]. Authors found that high heating rates are extremely important to produce well-structured and uniform materials. Time elongation produced small shrinkages of the structure. Regarding heating time, an aging of 3 h is enough to obtain well-defined pore sizes.

By using the same phenyl-silylated reagent, propylsulfonic acid-functionalized mesoporous benzene-silica (Ph-PMO-SO_3_H) was synthesized [70]. As conventional synthesis of this PMO required very long synthesis times (80–100 h), a microwave-assisted synthetic route was developed. In this way, the synthesis time could be reduced by an 80% (12 h). Several self-assembly and hydrothermal times under microwave radiation were studied, and the structural characteristics of the final solid measured. MW enhanced condensation reactions and thus Si–O–Si bonds, but excessive radiation decreased the structural order. In any case, MW produced faster reaction times, and aggregated particles with higher content of acid sulfonic groups.

Hybrid organic–inorganic materials can be also prepared by post synthesis grafting by using different trialcoxysilanes depending on the desired functionality on the silica surface. In this way, Egues et al. reported a couple of one-pot methods to obtain silica nanoparticles functionalized with different groups [71,72]. Silica nanoparticles were synthesized by irradiating a TEOS solution in mixed ethanol-water media for 1 h at 40 °C. Then, particles were placed back into the microwave and functionalized with 3-chloropropyl-trimethoxysilane. Additionally, the solids were further functionalized by the nucleophilic attack of 1-methylimidazole [72]. Although neither discussion nor characterization of the raw silica particles was provided, authors studied the time and temperature as parameters affecting the functionalization step. In general, higher reaction temperatures produced increased specific surface areas.

A summary of the literature discussed in this Section 2.5 is shown in Table 3.

## 3. Microwave-Assisted Synthesis of Silica Non-Porous Materials

Hitherto, the applicability of microwaves to the quick and controlled production of silica mesoporous solids with miscellaneous applications has been evidenced. These uses are mainly related to the excellent properties of porous silica solids, like high surface areas or the controllable pore sizes, depending on the surfactant used as template and its concentration. However, applications of silica are not only limited to porous materials. As will be shown below, silica nanoparticles, porous or not, are a relevant technological platform due to their easily modifiable surface and their ability to modify the properties of host materials including them.

### 3.1. SiO_x_-C Materials for Electrical Applications

**Scheme 4:**
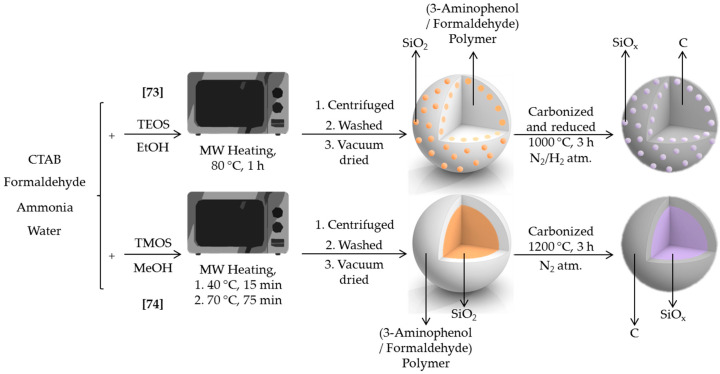
Synthesis scheme for the microwave assisted synthesis of C/SiO*_x_* nanocomposite [73] and SiO_x_@C [74].

Silicon derivatives have demonstrated to substantially improve the electrical properties of batteries. Either coating carbon as a film or as particles, these solids show improved conductive properties compared to precursor materials. Silicon oxides (SiO*_x_* (0 < *x* < 2)) act as semiconductors and provide lower volume change, and thus better durability, than silicon when introduced as anodes in lithium ion batteries. The main drawback of such substitution, the lower capacity delivered in batteries of SiO*_x_* in comparison with Si, can be overtaken adding a conductive layer like carbon or graphite. Taking this into account, Ogi et al. developed two microwave-assisted procedures to easily prepare carbon-SiO*_x_* particles for Li-ion battery anodes [73,74] (Scheme 4).

In the first case, SiO*_x_* nanoparticles were immobilized on carbon surface [73]. Two steps were required for this synthesis. In a first step, the surface of 3-aminophenol polymer was decorated with SiO_2_ particles, by placing a reaction mixture including TEOS, 3-aminophenol, CTAB, formaldehyde, ammonia, EtOH, and water in a microwave at 80 °C for 1 h. 3-aminophenol and formaldehyde were responsible of polymer formation, whereas the other reagents were related to formation of SiO_2_ nanoparticles according to Stöber process. In particular, the concentration of 3-aminophenol has been reported as the main factor to control the SiO_2_ particle size [75]. The 3-aminophenol polymer/SiO_2_ nanocomposite generated was simultaneously carbonized and reduced during a second synthesis stage, to produce the desired C/SiO*_x_* composite. The latter was fully electrochemically characterized, showing a 30% improvement compared to carbon-only electrodes.

In the second paper, the authors synthesized carbon-coated SiO*_x_* (SiO*_x_*@C) core–shell particles [74]. Again, two stages were required. In the first one, the reaction mixture of tetramethyl orthosilicate (TMOS), 3-aminophenol, CTAB, formaldehyde, ammonia, MeOH, and water was stirred for 15 min and then placed in a microwave at 70 °C for 75 min. As a result, SiO_2_@3-aminophenol-formaldehyde resin particles were formed. The core–shell particles were then carbonized at 1200 °C under N_2_ to obtain SiO*_x_*@C as highly uniform spherical particles. These core-shell particles were not formed in the absence of methanol or CTAB. According to the formation mechanism proposed, TMOS and MeOH diffused into the CTAB micelles and generate the core through the hydrolysis and condensation process. 3-aminophenol-formaldehyde resin would interact with the positively charged surface giving rise to the shell. Besides prominent advantages like mild reaction conditions, low cost, or high amount production, SiO*_x_*@C showed excellent properties when used as anodes in lithium-ion batteries, with capacity retention around 80% after 100 cycles.

Furthermore, carbon derivatives are well known for their excellent dielectric properties to absorb microwave radiation and to convert it into heat. Such a characteristic was used to achieve the coating of graphite flakes (GFs) with a layer of silicon derivatives [76]. Specifically, GFs were immersed in a mixture of siloxanes. When subjected to MW radiation, GFs selectively absorbed the radiation and reached high temperatures, inducing the SiO_2_-precursor molecules to deposit on these hot surfaces. As the promoted film is a bad microwave-absorber and heat conductor, the reactions on the surface automatically ceased by themselves. A final calcination step produced coating transformation into SiO_2_, with a thickness of 8 nm approximately. Therefore, 15 min of low power microwave radiation were able to produce high-performance composites, able to be used as anode materials for lithium-ion battery applications with fast charging capability, improved capacity, and excellent cycle stability.

In another approach, graphite nanoplatelets (GNPs) coated with silica nanoparticles were synthesized under microwave-assisted conditions [77]. Under a two-step procedure, GNPs were first placed in a solution with polyvinylpyrrolidone (PVP), which was adsorbed on the surface. Then, a basic pH was promoted by ammonia addition, causing pyrrolidone ring of PVP to be predominantly as enol form. Its hydroxyl groups reacted with TEOS in a microwave oven, leading to Si–O–C chemical bonds. In this way, in just 5 min, silica nanoparticle-coated GNPs were obtained. These 20–30 nm diameter nanoparticles were uniformly distributed on the surface. The formation mechanism was corroborated by repeating the procedure in the absence of PVP but, in this case, not a homogeneous nucleation of silica nanoparticles was formed on the GNPs but in the reaction medium. In conclusion, this simple and fast method provided Si-modified GNPs with an increased surface resistivity and improved dispersion stability in water compared to GNPs starting materials.

### 3.2. Analytical Applications

Silica particles can be also applied to analytical targets. In fact, high-performance liquid chromatography (HPLC) columns are packed with silica microspheres and, depending on their size and packing compression, the performance of the chromatography can be affected. When in the column, the mixture to separate must cross the column passing along the interstitial spaces between the silica spheres and interacting with the porosity and functional groups on the surface of the particles. As using smaller particles produce considerably increased back pressure, which put at rick instrumentation and operation, alternatives like sphere-on-sphere (SOS) silica particles emerged as an alternative to this drawback.

SOS silica particles can be prepared at room temperature in 24 h in a one-pot synthesis by only using 3-mercaptopropyltrimethoxysilane (MPTMS) as precursor [78]. However, a microwave method reported allowed its synthesis in just 5 min under irradiation at 40 °C [79]. Authors investigated different parameters affecting SOS synthesis: Si precursors, stirring (type and speed), temperature, and reaction time under microwave heating. As a result, uniform size-controlled SOS particles were obtained in shorter time and using low power radiation. 40 °C was selected as optimal, because higher temperatures produced other type of microspheres or aggregates. Using TEOS, TMOS, or (3-mercaptopropyl)triethoxysilane (MPTES), rather than or combined with MPTMS produced a gradual loss of the morphology. SOS particles obtained where modified with thiols or C_8_ functional groups, and their performance was successfully tested on sugars or TM2 test mixture (containing benzamide, acetophenone, benzophenone, and biphenyl) separation respectively.

### 3.3. No Reported Applications

Despite all the great application described until now for silica materials and composites, most of the reported literature regarding non-porous silica materials set the basis of the synthesis and characterization, and study how the synthesis parameters influence each other with regard to final material features. Here below, articles referring to silica particle synthesis or silica shells prepared using microwave technology will be presented.

The group of Luzinov et al. studied the influence of different synthesis parameters including the effect of microwaves on the final features of silica nanoparticles. In two closely related articles, they depict a microwave based synthetic method [80,81]. In particular, the authors use a mixture of tetramethyl orthosilicate (TMOS) and HCl solved in acetone. Acetone is a low loss-factor solvent, so it minimally couples with the electromagnetic field and allows the efficient and selective microwave absorption by the reactants. On the other hand, as the silicon precursor must be soluble in acetone, they used a mixture of tetramethyl orthosilicate (TMOS) in HCl. Acid produced two effects. It generates the silicic acid precursor, which is soluble in acetone and, also reduces condensation reaction rate. In such a way, different silicic acid:acetone ratios were placed in a closed microwave vial and heated to 125 °C for 1 min. Spherical silica nanoparticles as small as 30 nm (± 5 nm) or as large as 250 nm (± 30 nm) were synthesized in controlled manner by simply varying the concentration of silicic acid precursor and the duration of MW irradiation. In general, higher TMOS concentrations promoted larger particle sizes. Regarding microwaves, longer irradiation times produced larger particles, with minimal effects for longer times than 1 min. Moreover, increasing reaction times produced more spherical shapes. In any case, and compared to traditional Stöber particles prepared in basic media, the reported particles showed roughened surface morphology, caused by the acid media.

#### Coating with Silica

In some cases, particles are not fully made of silica, and silica is just used as a shell to protect the inner content from air or degradation, to improve their biocompatibility or for allowing an easier surface modification. Thanks to the fast and homogeneous heating produced by microwaves, they have been used to protect a wide range of cores with silica. In such a way, magnetic nanoparticles (MN, magnetite and/or maghemite) were coated with silica [82]. Magnetic nanoparticles covered with oleylamine were transferred to aqueous media with tetramethylammonium hydroxide and different amounts on TEOS under microwave irradiation. Particles aggregated as a consequence of phase transfer and, after irradiating for 10 min at 70 °C, an amorphous coating of silica covered the magnetic nanoparticles, producing the encapsulation of multiple cores. The higher the amount of TEOS, the less aggregated the particles and the thicker the spherical shell formed. MN@SiO_2_ nanocomposites produced in such a way were stable in aqueous solution and kept their superparamagnetic characteristics at room temperature.

Similarly, silver nanoparticles stabilized with oleylamine can be coated with silica to produce Ag@SiO_2_ core–shell nanostructures [83]. Authors observed that the addition of sodium citrate was necessary in order to remove remained Ag(I) ions from silver nanoparticles synthesis, because these ions produced multicore Ag@SiO_2_ nanoparticles. In this way, the addition of TEOS to the starting solution of silver nanoparticles in the presence of sodium citrate produced Ag@SiO_2_ in 10 min under a microwave-assisted method. An increase in the amount of TEOS produced a growth in the silica shell thickness. Independently of the initial silver nanoparticle size, the thickness of the shell was very uniform, in the range 20–30 nm. The great uniformity observed was ascribed to oleylamine, which linked SiO_2_ to the surface of Ag.

**Figure 1:**
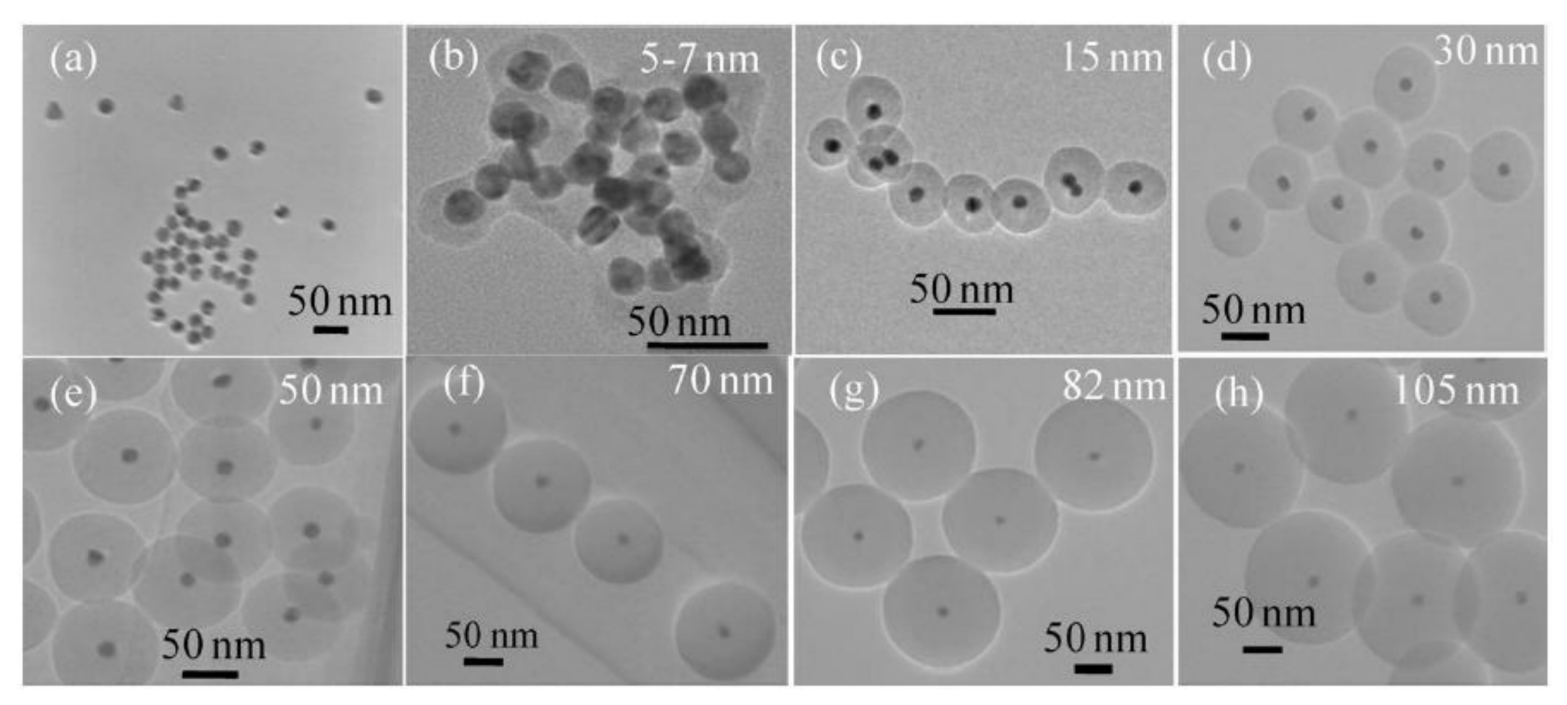
TEM images of silica encapsulated gold nanoparticles. Different silica shell thicknesses were produced by MAS when concentration of TEOS raised from none (**a**), 1 mM (**b**), 2 mM (**c**), 3 mM (**d**), 5 mM (**e**), 10 mM (**f**), 15 mM (**g**), up to 20 mM (**h**). Reproduced with permission from [84]. Elsevier, 2011.

Gold nanoparticles have also been coated with silica following a microwave-assisted method [84]. First, citrate-stabilized gold nanoparticles were prepared under MW radiation, and then, they were further coated with a silica shell by the addition of TEOS. 5 min of microwave irradiation at 50 °C were found to be optimal for the synthesis, in order to predominantly produce single core Au@SiO_2_ nanoparticles and avoid Au aggregates or core free SiO_2_ particles. By simply varying the amount of TEOS used, the silica shell thickness could be tuned in a wide range, from 5 to 105 nm (Figure 1). The produced coating was more uniform and the nanoparticles were more monodisperse than those prepared under conventional conditions, thanks to the uniform heating produced by microwaves. Moreover, particles were functionalized in the microwave with amino, carboxylate, and alkyl groups, in order to provide them of a better functionality for biological applications.

Metal oxides like zirconium dioxide (ZrO_2_) can be coated with silica. Thanks to the application of microwaves, synthesis times can be notably reduced to a few minutes, compared to time wasting conventional conditions that need times around 24 h [85]. A microwave-assisted method for the synthesis of silica-coated ZrO_2_ nanoparticles was developed [86]. In only 2 min, a uniform shell of SiO_2_ with a thickness of about 2 nm was formed over ZrO_2_ particles, thick enough to totally hide the underlying zirconia. ZrO_2_ thickness can be easily controlled by varying the amount of TEOS added to the mixture, which gave very fast hydrolysis and polymerization on ZrO_2_ surface, finally coating the particles.

Not only metallic nanoparticles but also covalent wires of SiC can be coated with silica [87]. Thanks to microwaves, a mixture of Si, SiO_2_, and charcoal could be heated to over 1300 °C in only 10 s. After keeping constant the conditions for 20 min, micrometrical SiC nanowires encapsulated in a SiO_2_ shell were formed. The lengths of the wires were in the range of several tens to several hundreds of micrometers, whereas they just were 10–50 nm in diameter. Moreover, wires were highly aligned, and core was formed of multiple SiC. Thanks to silica shell, characteristic SiC emission was blue-shifted, and nanowires emitted a stable and intense violet-blue light when excited under 325 nm radiation.

A summary of the literature discussed in Section 3 is shown in Table 4.

## 4. Summary and Expectations

In conclusion, although silica solids have been used from several decades up to now, they are still subject matter. The vast knowledge derived from their study and their unique properties make them the perfect tool to found new or improved applications. Moreover, the interaction with electromagnetic fields, and their certain effects on the final materials, has opened up new ways of preparing materials, and new applications for them. Some of the general benefits of using microwaves as source of energy are faster reaction, higher reproducibility, and the possibility to obtain materials with differentiated properties. When applied to the synthesis of silica-based materials, the reviewed articles have shown improved physicochemical features (such size, shape, and porosity), narrower particle size distribution and shorter reaction times. This field is, in fact, a promising way to find remarkable and new uses to silica-derived materials, and this review has tried to report the latest and most remarkable applications of microwave-assisted produced silica-based solids.

Despite the numerous examples of silica materials included in this review and in previous revisions, there are diverse research opportunities that would improve the relevance of the discipline. For many years, microwaves have been used for the synthesis of silica materials empirically, driven by the general benefits of microwaves in solvothermal conditions listed above. However, the rational design of materials and synthetic procedures is still a challenge that requires of a deeper theoretical background.

The interaction of the electromagnetic field with each molecule in solution and material differs and, therefore, an evolution in the solution properties can be expected during the hydrolysis-condensation process. Furthermore, we can expect different behaviors depending on the dielectric properties of the solvent and the microwave absorption capacities with the chemical nature, size, and topology of the materials. That can generate a preferential heating of some materials. The analysis and modelization of such behaviors require of basic research and the interaction of multidisciplinary groups specialized in the chemical, materials, and physics area.

A deeper description of the synthesis conditions would be advisable. It would improve the reproducibility and the processes design. In several cases, the methods and equipment are barely described. Clear recommendations for the content of the experimental procedures would benefit the extension of the discipline and its adoption by other groups.

In comparison to the conventional heating methods, the microwave oven is a key element. Even now, several papers are published using household microwave ovens with very little control of the conditions. Professional instruments based in magnetrons as source of microwaves offer a better control, but they are quite expensive. By contrast, in the recent years, solid state generators of microwaves have emerged as an incipient alternative for cheaper reactors with high control and long duration. This kind of technology will improve the synthesis of silica materials with microwaves and broad their presence in the chemical laboratories. We will start to see such benefits as these kinds of generators are included in the commercial reactors and their possibilities are available to materials scientists

We expect that this review will bring the scientific community closer to the possibilities and benefits of using microwaves as a standard tool in the synthesis of silica materials.

## Figures and Tables

**Table 1:** Synthesis parameters and characterization of the solids prepared for waste removal applications.

Ref.	Solid	BET (m^2^ g^−1^)	Pore Size (nm)	Particle Size	Si Source	Surf.	Temp. (°C)	Power (W)	Time (min)
[41]	SSBA-15	573	9.97	450 ± 50^a^ nm	TEOS	P123	n.a.	n.a.	120
[42]	SBA-15-NH_2_	128	n.a.	n.a.	TEOS	P123	80	n.a.	420
[43]	Ti-SiO_2_	n.a.	3.40	0.1–20^a^ µm	TEOS	STAC	60	n.a.	30
[44]	SiO_2_-TiO_2_	653	2.15	1.3^a^ µm	TEOS	CTAB	n.a.	120	90
[45]	Ti-MCM-41	933	2.81	4–8^a^ µm	TEOS	CTAB	n.a.	210	40
[46]	CeO_2_-SiO_2_	335	n.a.	8^b^	TEOS	CTAB	160		30
[47]	PMO	493	8.70	n.a.	SS	P123	100	300	120
[48]	Fe_3_O_4_-SiO_2_	308	6.14	4.7–9.1^b^ nm	SS	None	80	n.a.	10
[49]	Fe_3_O_4_-SiO_2_	n.a.	n.a.	n.a.	TEOS	CTAB	15	n.a.	90
[50]	MCM-41	1254	3.60	n.a.	SF	CTAB	n.a.	n.a.	40
[51]	MCM-41-NH_2_	258	3.25	n.a.	FA	CTAB	n.a.	n.a.	30
[52]	SiO_2_	204	21	16–28^b^ nm	Sand	None	n.a.	100	0.5
[53]	SiO_2_	85	9.84	93^a^ nm	RH	None	80	n.a.	30

BET: Brunauer-Emmett-Teller surface area; Surf.: surfactant; Temp.: temperature; a: measured in SEM; b: measured in TEM; n.a.: information not available; TEOS: tetraethyl orthosilicate; STAC: stearyltrimethylammonium chloride; CTAB: cetyltrimethylammonium bromide; P123: pluronic 123; SS: sodium silicate; SF: silica fume; FA: fly ash; RH: rice husk.

**Table 2:** Synthesis parameters and characterization of the solids prepared for catalysis, drug release and gas adsorption applications

Ref.	Solid	BET (m^2^ g^−1^)	Pore Size (nm)	Particle Size (nm)	Si Source	Surf.	Temp. (°C)	Power (W)	Time (min)
[54]	Proline-SiO_2_	727	5.60	n.a.	SS	P123	100	300	120
[55]	Ti-SBA-15	718	5.41	n.a.	TEOS	P123	90	200	60
[56]	Ti-MCM-41	882	2.80	n.a.	TEOS	CTAB	120	n.a.	40
[57]	SiO_2_	817	3.13	30−45^b^	TEOS	CTAB	n.a.	450	60
[58]	Pd-MCM-41	1516	2.49	n.a.	SS	CTAB	n.a.	120	30

BET: Brunauer-Emmett-Teller surface area; Surf.: surfactant; Temp.: temperature; b: measured in TEM; n.a.: information not available; SS: sodium silicate; P123: Pluronic P123; TEOS: tetraethyl orthosilicate; CTAB: cetyltrimethylammonium bromide.

**Table 3:** Synthesis parameters and characterization of the mesoporous solids prepared under microwave irradiation

Ref.	Solid	BET (m^2^ g^−1^)	Pore Size (nm)	Particle Size (nm)	Si Source	Surf.	Temp. (°C)	Power (W)	Time (min)
[60]	MCM-41	1138	3.21	n.a.	SG	CTAB	100	1200	90
[61]	MCM-41	1438	4.00	70^a^	SS	CTAB	120	120	30
[62]	MCM-41	1138	n.a.	n.a.	SG	CTAB	100	1200	90
[63]	MCM-41	980	3.70	n.a.	SS	CTAT	190	1450	2
[64]	SBA-15	900	6.50	n.a.	TEOS	P123	90	n.a.	120
[65]	SBA-16	866	9.00	n.a.	TEOS	F127	100	n.a.	300
[66]	FDU-1	622	8.50	n.a.	TEOS	V504	100	n.a.	60
[67]	Zr-SiO_2_	830	2.47	n.a.	SS	CTAB	Reflux	220	150
[68]	P-SBA-15	705	8.5	n.a.	SS	P123	100	n.a.	600
[69]	PMO	957	2.40	n.a.	BTEB	CTAB	100	400	180
[70]	PMO	857	3.15	629^a^	BTEB / MPTMS	STAB	100	1200	180
[71,72]	SiO_2_-Cl SiO_2_-Im	n.a.	n.a.	n.a.	TEOS	None	40	300	60

BET: Brunauer-Emmett-Teller surface area; Surf.: surfactant; Temp.: temperature; a: measured in SEM; b: measured in TEM; n.a.: information not available; SG: silica gel; CTAB: cetyltrimethylammonium bromide; SS: sodium silicate; CTAT: cetyltrimethylammonium tosylate; TEOS: tetraethyl orthosilicate; V504: vorasurf 504; BTEB: 1,4-bis(triethoxysilyl)benzene; MPTMS: (3-mercaptopropyl)trimethoxysilane; STAB: stearyltrimethylammonium bromide; P123: Poly(ethylene glycol)-block-poly(propylene glycol)-block-poly(ethylene glycol); F127: Poly(ethylene glycol)-block-poly(propylene glycol)-block-poly(ethylene glycol) diacrylate.

**Table 4:** Synthesis parameters and characterization of the non-porous silica solids prepared under microwave irradiation.

Ref.	Solid	Particle Size	Si Source	Temp. (°C)	Power (W)	Time (min)
[73]	C/SiO_x_	n.a.	TEOS	80	n.a.	60
[74]	SiO_x_@C	n.a.	TMOS	40	n.a.	15
[76]	GF@SiO_2_	8^b^ nm	Siloxanes	n.a.	300	15
[77]	SiO_2_-GNP	20–30^a^ nm	TEOS	70	1000	5
[79]	SOS	5^a^ µm	MPTMS	40	200	5
[80,81]	SiO_2_	n.a.	TMOS	125	65	1
[82]	MN@SiO_2_	131–167^b^ nm	TEOS	70	200	10
[83]	Ag@SiO_2_	50–200^b^ nm	TEOS	n.a.	360	10
[84]	Au@SiO_2_	5–105^b^ nm	TEOS	50	n.a.	5
[86]	ZrO_2_@SiO_2_	2^b^ nm	TEOS	70	n.a.	2
[87]	SiC@SiO_2_	n.a.	Si, SiO_2_	n.a.	800	20

Temp.: temperature; a: measured in SEM; b: measured in TEM; n.a.: information not available; TEOS: tetraethyl orthosilicate; TMOS: tetramethyl orthosilicate; GF: grafite flake; GNP: graphite nanoplatelet; SOS: sphere-on-sphere; MPTMS: (3-mercaptopropyl)trimethoxysilane; MN: magnetic nanoparticle.
